# Cutaneous leishmaniasis situation analysis in the Islamic Republic of Iran in preparation for an elimination plan

**DOI:** 10.3389/fpubh.2023.1091709

**Published:** 2023-04-28

**Authors:** Iraj Sharifi, Ahmad Khosravi, Mohammad Reza Aflatoonian, Ehsan Salarkia, Mehdi Bamorovat, Ali Karamoozian, Mahmoud Nekoei Moghadam, Fatemeh Sharifi, Abbas Aghaei Afshar, Setareh Agha Kuchak Afshari, Faranak Gharachorloo, Mohammad Reza Shirzadi, Behzad Amiri, Mohammad Zainali, Sara Doosti, Omid Zamani, Mohammad Mahdi Gouya

**Affiliations:** ^1^Leishmaniasis Research Center, Kerman University of Medical Sciences, Kerman, Iran; ^2^Research Center for Modeling in Health, Institute for Futures Studies in Health, Kerman University of Medical Sciences, Kerman, Iran; ^3^Research Center for Health Services Management, Kerman University of Medical Sciences, Kerman, Iran; ^4^Research Center of Tropical and Infectious Diseases, Kerman University of Medical Sciences, Kerman, Iran; ^5^Medical Mycology and Bacteriology Research Center, Kerman University of Medical Sciences, Kerman, Iran; ^6^Center for Communicable Diseases Control, Ministry of Health and Medical Education, Tehran, Iran; ^7^Universal Health Coverage for Communicable Diseases (UHC: CD), World Health Organization, Country Office, Tehran, Iran

**Keywords:** cutaneous leishmaniasis, situation analysis, prevention, elimination plan, Iran

## Abstract

Iran has invariably been under the growing public health threat of cutaneous leishmaniasis (CL), a significant barrier to local development that hinders the prevention and control efforts toward eliminating the disease. So far, no comprehensive and in-depth epidemiological analysis of the CL situation has been carried out nationwide. This study aimed to employ advanced statistical models to analyze the data collected through the Center for Diseases Control and Prevention of Communicable Diseases during 1989–2020. However, we emphasized the current trends, 2013–2020, to study temporal and spatial CL patterns. In the country, the epidemiology of CL is incredibly intricate due to various factors. This fact indicates that the basic infrastructure, the preceding supports, and the implementation plan related to preventive and therapeutic measures need crucial support. The leishmaniasis situation analysis is consistent with desperate requirements for efficient information on the control program in the area. This review provides evidence of temporally regressive and spatially expanding incidence of CL with characteristic geographical patterns and disease hotspots, signifying an urgent need for comprehensive control strategies. This information could be a suitable model and practical experience in the Eastern Mediterranean Region, where over 80% of CL is reported.

## 1. Introduction

Leishmaniasis is among the most emerging and re-emerging zoonotic diseases that have long been recognized, evolved, remarkably increased, and gradually expanded beyond the traditional geographical range and progressively dispersed among different hosts and numerous vectors (1). This disease frequently occurs, spreading greater and faster than before (2). In 2018, over 253,000 new cutaneous leishmaniasis (CL) cases were reported to World Health Organization (WHO). The region accounts for over 80% of the CL caseloads worldwide, only from the Eastern Mediterranean Region (EMR) countries, representing a primary “hotspot” eco-epidemiological region (3). Cutaneous leishmaniasis is a multidimensional entity posing a severe public health concern in the WHO EMR in which 18/22 (82%) of countries and territories were endemic for CL. Several epidemiological and clinical forms indicate a challenge in managing, controlling, and eliminating the disease (4). Leishmaniasis is still the third most vector-borne disease and is frequently fatal if it remains untreated in 101 tropical and subtropical countries and territories (5, 6). Although visceral leishmaniasis (VL or kala-azar) is the deadliest type, CL is the most common form encompassing three-fourths of the total burden (7).

Over the past three decades, despite tremendous efforts to control CL in Iran, it seems that the burden of the disease is still high in some areas. This fact indicates that the basic infrastructure and the preceding support and implementation plan related to diagnostics, drugs, and vaccines are inadequate nationally and abroad (8). The leishmaniasis situation analysis and the suggested framework is consistent with desperate requirements for efficient information on the range of the control program in the area as previously outlined by the WHO Resolution (9). This resolution called for conditions that would allow WHO to assume a leadership role in scientific and technical cooperation to strengthen, initiate, maintain, and expand the leishmaniasis control program. The outcome has been streamlined, and integrated approaches have produced extraordinary gains for public health improvement. Iran is among the forefront affected countries that, together with Brazil, Peru, Columbia, Algeria, Afghanistan, Syria, and Pakistan, constitute the eight most endemic countries (5). Like the Old World, *Leishmania major* and *L. tropica* are responsible for zoonotic (ZCL) and anthroponotic CL (ACL) in Iran, respectively (10). In the country, the epidemiology of CL is incredibly intricate due to the complexity of the rural and urban life cycles, the implication of numerous reservoir hosts and varying species of phlebotomine sandfly vectors, variable response to standard medications, complex confounding factors, mixed species circulating in the endemic foci, various clinical features and recurrent emerging epidemics and main challenges and gaps (1, 11–14).

No similar comprehensive and in-depth epidemiological analysis of the CL situation has been carried out at the national level. This study analyzed the national data collected through the Center for Diseases Control and Prevention of Communicable Diseases (CDC) during 1989–2020. However, we emphasized the current trends, 2013–2020, to study temporal and spatial CL trends and pinpoint hotspots. Identification of the persistent and high-incidence areas could help the program prepare an elimination plan for future control planning, as requested by the CDC, Ministry of Health and Medical Education (MOHME), and WHO. Herein, we provide evidence of temporally regressive and spatially expanding incidence of CL with characteristic geographical patterns and disease hotspots, signifying an urgent need for intensified, effective, and comprehensive control strategies, particularly in the hotspot settings. The information presented in this study could be a suitable model and practical experience in EMR countries and worldwide.

## 2. Data sources and scope

The primary data source was those reported monthly by the health surveillance system to the CDC in the country and via literature review in the area of interest relevant to the characteristics of the CL cases. We have presented the current situation of CL and reviewed various operational aspects of the current and preceding control strategies, including implementing prophylactic and therapeutic measures in humans, animal reservoirs, and sandfly vectors. We have likewise summarized significant challenges and gaps, present status, and finally listed a framework of strategic approaches and essential needs for conducting a preliminary control program toward a CL elimination plan. Furthermore, a leishmaniasis expert team comprising experienced staff and academic members affiliated with the universities at the provincial and national levels was established. During a 24-month work, 23 meetings were held together with a literature review, interviews, visits, and discussion of various leishmaniasis issues, particularly those which might interfere with the future control program. Furthermore, relevant challenges and gaps analyses linked to strengths, weaknesses, opportunities, and threats (SWOT), and a list of essential needs were presented.

## 3. Geographical areas

Iran consists of 31 provinces, 85 million population, and 1,648,000 km^2^ areas. This country possesses one of the longest land borders (5,894 km) of any country in western Asia, neighboring Afghanistan, Pakistan, Turkey, Armenia, Azerbaijan, Turkmenistan, and Iraq. Iran is a vast country with inconstant and different climates: mild and relatively wet on the coast of the Caspian Sea, continental and arid in the plateau, cold in high mountains, and desert, and hot on the southern coast in the southeast. In the northwest, winters are cold with heavy snowfall and subfreezing temperatures. Spring and fall are relatively mild, while summers are dry and hot. In the south, winters are mild, and the summers are sweltering, having average daily temperatures in July exceeding 38°C. On the Khuzestan plains, the summer heat is accompanied by high humidity (15, 16). In general, Iran has a continental climate where most of the relatively scant annual precipitation falls from October through April (17). Such a diverse range of temperatures, climatic zones, and fertile land creates an opportunity for growing all kinds of crops and vegetables. These conditions brought about fertile grounds for providing multiple risk determinants and suitable breeding environments for propagating sandflies and reservoirs, contributing to an excessive number of CL cases in a geographically vast area of the country (18, 19).

## 4. Epidemiological characteristics

### 4.1. Case–definition

A confirmed CL case refers to a patient who shows clinical signs (skin lesions) along with parasitological confirmation of the organism (positive smear or culture obtained from the edge of the skin lesion). The confirmed CL cases are routinely diagnosed to the genus level (20); however, the investigators seeking research purposes are exceptions for those identified to the species phenotype.

### 4.2. Surveillance and case detection

Cutaneous leishmaniasis is a notifiable disease in Iran and is integrated into the primary health surveillance system (PHC). The CL cases are reported monthly from peripheral areas to district-province-national levels. In routine detection, passive case finding consists of screening for CL at health clinics, and rural or urban centers, while in emergencies, passive recognition, and active case detection of CL cases through house-to-house visits are carried out.

### 4.3. Clinico-epidemiological forms

Two main CL types are present in Iran, either alone or mixed, depending upon the endemic locality (21). Zoonotic CL (ZCL) is the most predominant and widespread form caused by *L. major*. The *Phlebotomus papatasi* female sandflies primarily transmit this species from small gerbils to humans, presumably in 80% of the foci (22) in 842 districts inhabiting 2.4 million at-risk populations. This form is endemic in many rural and municipal areas of 19 out of the 31 provinces of Iran, including Tehran, Isfahan, Kashan, Qom, Semnan, Khorasan Razavi, Khorasan Shomali, Golestan, Khorasan Jonoobi, Fars, Ilam, Lorestan, Bushehr, Khuzestan, Hormozgan, Kerman, Kermanshah, Yazd, and Sistan/Baluchestan (10, 23). The global foci of ZCL are vast, including numerous geographical areas in the Middle East, north-western China, and North Africa (10, 24). While anthroponotic CL (ACL) is caused by *L. tropica* and transmitted by the female *Ph. sergenti* sandflies from human to human, often in an urban life cycle. This form is the minor type (nearly 20%) present, more likely in 205 large and medium-sized cities and suburban settlements entailing Mashhad, Neyshbour, Sabzevar., Kashan, Yazd, Isfahan, Kerman, Bam, and Shiraz, within the country (20).

### 4.4. Reservoir hosts

Four species of small rodents belonging to the family Cricetidae are designated as the principal reservoir hosts for ZCL in several parts of Iran. *Rhombomys opimus* (the great gerbil) is the primary host of ZCL in central and northeast Iran, while *Meriones libycus* (the Libyan Jird) is the primary reservoir host of ZCL in the central and south of the country. *Tatera indicia* (the Indian gerbil) is the primary reservoir host of ZCL in the southeast of Iran. *Meriones hurrianae* (the desert gerbil) is implicated as the reservoir host of ZCL in Baluchistan, in southeastern Iran (25), while prominent infection has been described in *Nesokia indica* as well. Summary of presence probabilities for three main species (e.g., *R. opimus*, *M. libycus*, and *T. indica*) exposed to favorable environmental niches in widespread areas of 16/31 provinces (26–31). Anthroponotic CL caused by *L. tropica,* is restricted to humans, although sporadic dogs have been infected in some foci. It is assumed that dogs might be implicated in the epidemiology of the disease and play a secondary role in transmitting the *L. tropica* parasite (32, 33).

### 4.5. Biological vectors

Only a marginal of sandflies are biological vectors of CL in Iran. *Phlebotomus papatasi* is the primary and well-known sandfly vector of ZCL among humans and gerbils. Natural *L. major* promastigote infection has been detected in 0.2–22% of *Ph. papatasi* from rodent burrows. Some reports indicate that *Ph. salehi* is the secondary vector transmitting ZCL infections in an endemic focus of ZCL in central Iran (34–46). In contrast, ACL is found in long-lasting foci in medium and large-sized cities in highland areas. The extent of this disease was significantly influenced by anti-malaria insecticide spraying over the past decades. *Ph. sergenti* is the primary sandfly vector of ACL transmitting the organism in the endemic foci of Iran (25, 47–49).

### 4.6. Demographic and clinical features

We collected the CL new cases data (n = 695,541) between April 1983 and March 2020. Subsequently, a subsample (n = 129,009) from April 2013 to April 2020 was used by advanced statistical analyses. The CL patients consisted of males (58%) and females (41%) aged 1–83 years old who were interviewed, medically examined, and diagnosed for the presence of active skin lesions or scars compatible with CL by the health networks in 31 provinces of the Islamic Republic of Iran. There was a significant difference between males and females (*p* < 0.001). All age groups were affected, but children <10 years old performed the highest (23%), and > 60 years the minor level of infection (8%) (*p* < 0.001). Most of the patients lived in urban areas (52%), the majority were Iranian (93%), and the remaining (7%) Afghan migrants. Most of the lesions were single (47%), 1 cm in diameter (46%), and frequently on hands (57%).

**Figure 1:**
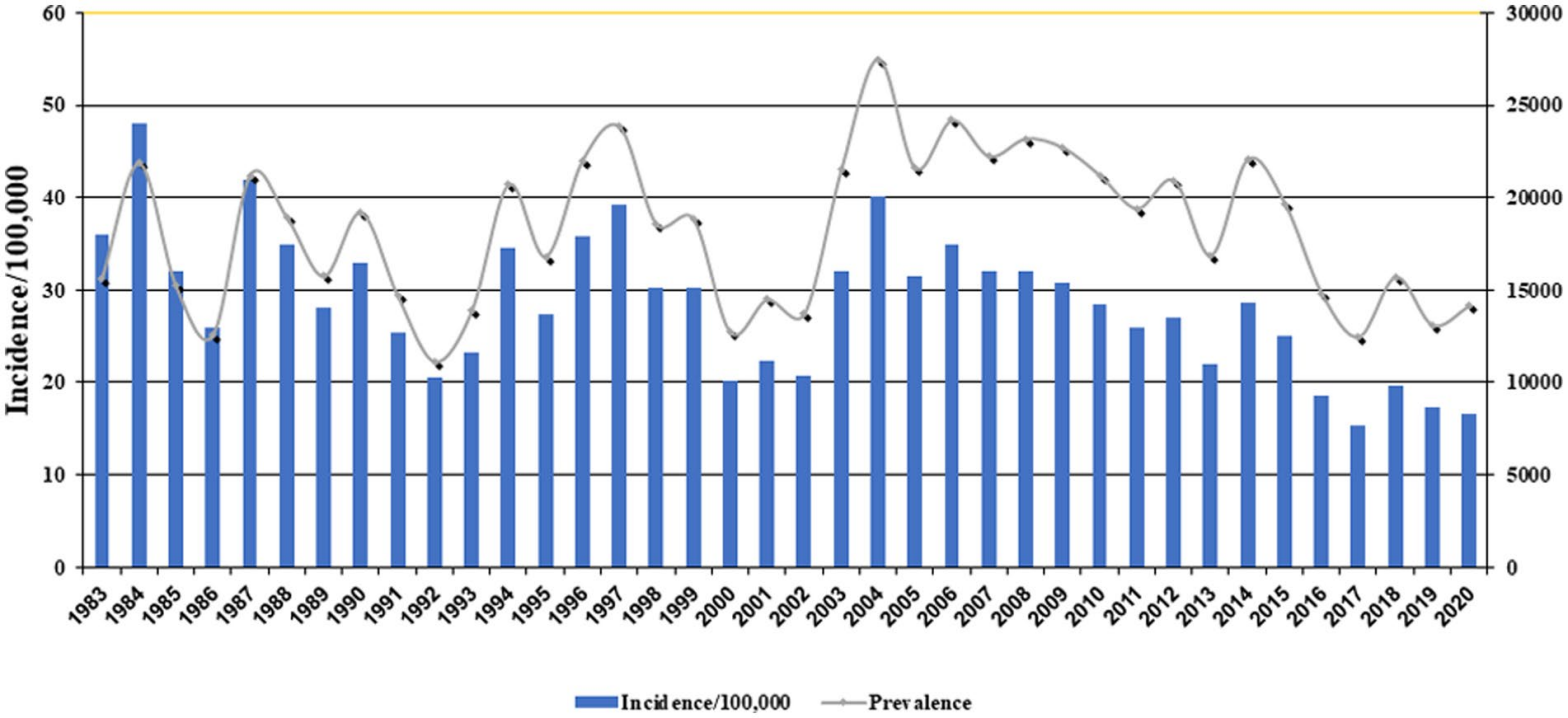
Incidence and prevalence rates of cutaneous leishmaniasis in Iran, 1983–2020.

Figure 1 exhibits the nationwide CL prevalence and incidence of cases during 1983–2020. Over this period, the trend was somewhat sluggish, but more or less of fluctuating nature and steadily declined. This variation was mainly due to multiple risk factors, especially earthquakes, natural and anthropogenic environmental changes, population displacement, and drug unresponsiveness (50–52). The five provinces with the highest incidence were Ilam 143, Fars 75.3, Semnan 60.2, Isfahan 48.8, and Golestan 46 per 100,000 people. The average incidence rate was 20.7 per 100,000 persons nationally (Table 1).

**Table 1:** Annual incidence (per 100,000 population) trends of cutaneous leishmaniasis in various provinces of Iran, 2013–2020.

Provinces	2013	2014	2015	2016	2017	2018	2019	2020	Average
Ilam	102.6	271.5	224.1	180.6	135.0	77.0	78.7	74.6	143.0
Fars	86.7	112.7	123.6	74.1	57.0	59.7	40.0	48.8	75.3
Semnan	28.7	30.4	29.7	48.5	30.9	41.4	95.9	175.8	60.2
Isfahan	60.3	59.7	38.1	28.1	22.9	68.3	59.1	53.6	48.8
Golestan	32.9	36.2	43.6	51.4	33.3	75.7	60.9	33.9	46.0
Khuzestan	19.2	80.5	60.4	34.9	31.0	29.7	20.7	36.1	39.1
Khorasan Razavi	63.9	53.3	42.7	40.5	34.5	26.4	23.4	15.4	37.5
Khorasan Shomali	50.7	55.5	31.1	22.6	24.8	35.5	24.0	20.0	33.0
Yazd	27.3	33.2	41.8	26.9	16.4	36.6	21.4	32.3	29.5
Kerman	41.6	32.4	25.9	25.8	23.8	22.2	17.1	13.6	25.3
Qom	18.8	15.3	13.4	1.8	11.8	25.7	21.3	19.6	16.0
Sistan and Baluchestan	13.6	15.2	9.5	6.9	5.2	6.7	12.0	18.6	11.0
Kermanshah	3.5	15.5	2.4	13.2	17.9	10.3	5.9	17.9	10.8
Lorestan	5.9	13.3	17.8	9.2	7.7	5.5	3.8	6.7	8.8
Bushehr	11.1	12.9	12.1	7.8	5.1	9.3	2.3	3.8	8.1
Kohkooliye &Boyerahmad	7.8	12.0	9.8	9.8	7.2	6.1	6.7	4.9	8.0
Hamedan	2.8	9.3	12.2	8.1	9.5	6.9	4.5	5.5	7.3
Khorasan Jonoobi	6.9	8.7	13.5	8.1	3.5	9.0	4.8	3.8	7.3
Chahar Mahal and Bakhtiari	7.4	9.5	9.1	4.7	3.6	7.6	6.1	7.7	7.0
Hormozgan	6.4	4.7	4.9	5.7	9.1	7.6	8.4	4.3	6.4
Tehran	1.9	2.2	2.2	2.8	2.3	3.9	3.1	3.3	2.7
Markazi	1.5	3.0	1.8	2.6	2.8	4.1	2.2	3.7	2.7
Alborz	1.1	2.1	2.0	1.9	1.7	2.0	2.4	2.5	2.0
Mazandaran	1.5	1.4	1.2	1.8	1.4	1.4	1.6	2.2	1.6
Ardabil	1.4	2.0	1.2	1.6	2.0	1.9	0.6	1.5	1.5
Ghazvin	1.1	0.7	0.5	0.5	2.9	2.1	1.3	1.4	1.3
Kordestan	0.5	1.6	2.4	1.5	2.4	0.7	0.4	0.6	1.3
East Azerbaijan	0.8	1.3	1.4	1.3	1.1	1.2	1.3	1.4	1.2
West Azerbaijan	0.4	0.9	1.1	0.6	1.1	0.8	1.0	1.0	0.9
Zanjan	0.5	0.5	0.5	1.1	1.0	1.2	0.5	0.7	0.7
Gilan	0.1	0.6	0.3	0.2	0.4	0.2	0.8	0.1	0.3
Average	19.6	28.9	25.1	20.1	16.4	18.9	17.1	19.8	20.7

Over the last decade, CL has expanded considerably, and numerous outbreaks and emerging epidemics of variable magnitudes occurred, attributed to the spread and resurgence of the disease and the provision of many confounding determinants (12, 14, 24, 53–56). Despite a significant reduction in VL cases, the geographical range of CL has remarkably enlarged. The policymakers, health authorities, and clinical practitioners have seriously overlooked the disease as they do not consider CL a severe threat and urgent public health concern in Iran and EMR. This is mainly due to the perceived no serious and under-reporting disease, other precipitation determinants, and many underlying chronic diseases (57). Iran is among the eight most affected and frontline countries where 15,000–20,000 cases of CL have officially been reported, although the actual number of new cases is reported to be in the order of 2-5-fold higher (20).

### 4.7. Analysis of national incidence trends

A non-linear mixed model (random effect model) was used to explore the incidence trend in different provinces and the effect of demographic data, including age and gender, on the incidence rate over time. To be precise, we employed ‘random intercept’ and ‘random slope’ in the above model. To assess the incidence trend based on different months and the nature of the non-linearity of the data, we used two models, including the ‘generalized additive model’ (GAM) and ‘generalized additive mixed model’ (GAMM). Finally, to predict the incidence trend for the following months and years, we used the ‘seasonal autoregressive integrated moving average (SARIMA) approach by $\mathrm{ARIMA}\;\left(2,0,0\right)\times {\left(2,1,0\right)}_{12}$. To analyze the various data, we performed R4.1.1 by using nlme, smooth, forecast, gam, gamm4, simts, and sarima packages.

The outcome of both effects, ‘fix’ and ‘random’ trends in the incidence of CL in different provinces, were significant (*p* < 0.001) at the national level (Table 2). This trend has decreased with time (estimate: −0.88, CI: −1.70, −0.05, and covariance between intercept and year, −0.48). We concluded that the provinces with a higher incidence rate, such as Ilam, Fars, Semnan, Isfahan, and Golestan, showed a faster-declining trend than the other provinces. Therefore, implementing the interventional measures recommended by WHO during 2014–2018 has effectively reduced the disease burden in high-risk provinces (58).

**Table 2:** The incidence trend of cutaneous leishmaniasis in various provinces of Iran by the fix and random effect models, 2013–2020.

Fix effect				
Parameters	Estimate	SE	value of *p*	%95 CI for estimate
Intercept	27.39	6.54	<0.001	(14.02, 40.77)
Year	-0.88	0.40	0.04	(−1.70, −0.05)
Random effect				
Parameters	Variance	value of *p*		Covariance between Intercept and Year
Intercept	42.86	<0.001		−0.48
Year	0.16	0.05		

**Figure 2:**
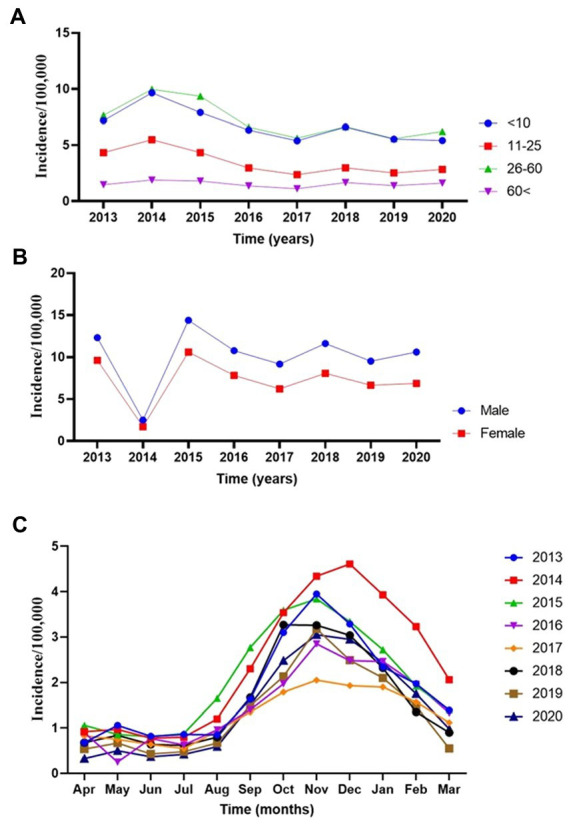
The annual incidence trend of cutaneous leishmaniasis in various provinces of Iran, 2013–2020. **(A)** Age-groups. **(B)** Sex. **(C)** Monthly.

Then we assessed the main effect of age and gender interactions with year on the incidence rate of CL (Table 3). There was a significant difference (*p* < 0.001) among <10 years old children and 10–25 or > 60 years old individuals. Similarly, there was a significant difference in the incidence of CL in males and females (*p* < 0.001) (Table 3). The annual trend of new cases by different age groups is presented in Figure 2. At the same time, if the interaction of age with year is being considered, there was only a significant difference (*p* < 0.03) between <10 and > 60 years older. No significant differences among <10 and other groups were observed (Figure 2A). Figure 2B displays the significance of the difference in incidence trend among males and females by time. There was a relatively constant trend in the incidence of CL in females and males from 2015 to 2020.

**Table 3:** Age and gender interactions of cutaneous leishmaniasis incidence trend in various provinces of Iran, 2013–2020.

Variable		Estimate	SE	Value of *p*
Age	<10	0		
	10–25	−3.10	0.69	<0.001
	25–60	1.27	0.69	0.07
	≥60	−5.27	0.69	<0.001
Sex	Male	0		
	Female	−3.44	0.97	<0.001
Year		−0.57	0.21	0.008
	(<10) with year	0		
Interactions of age with year	(10–25) with year	0.20	0.30	0.52
	(25–59) with year	0.16	0.30	0.59
	(≥60) with year	0.63	0.30	0.03

An equivalent number of affected males and females were described in public-based studies; however, investigations based on surveillance systems exposed a deceptive upsurge of males (51), possibly described by diverse clinical-seeking behaviors for both genders. A study in Iran confirmed that unresponsiveness to CL therapy is meaningfully linked with increasing age. The impairment of innate and adaptive immune systems may account for amplified drug unresponsiveness in older age groups (12). Male patients frequently do not complete the assigned medication, mainly because of insignificant treatment adherence. This partial adherence to treatment might play a critical role in treatment failure.

Then we explored the monthly trend in the incidence of the disease by the time of occurrence. Figure 2C exhibits seasonal trends reflecting non-linearity modeling tendencies. Incidence rates gradually declined during the spring and early summer from April to July, and a sharp upsurge from August to November during the cold months of the year was observed, where again, sharply decreased until March. It seems that the sandfly activities start sometime in the early months of spring (59) when the *Leishmania* agents undergo the incubation period. The CL lesions gradually appear in late summer, when a peak of CL occurs in August to November during the monsoon period. Meanwhile, the caseloads sharply dropped from January to March compared to the remaining months.

**Figure 3:**
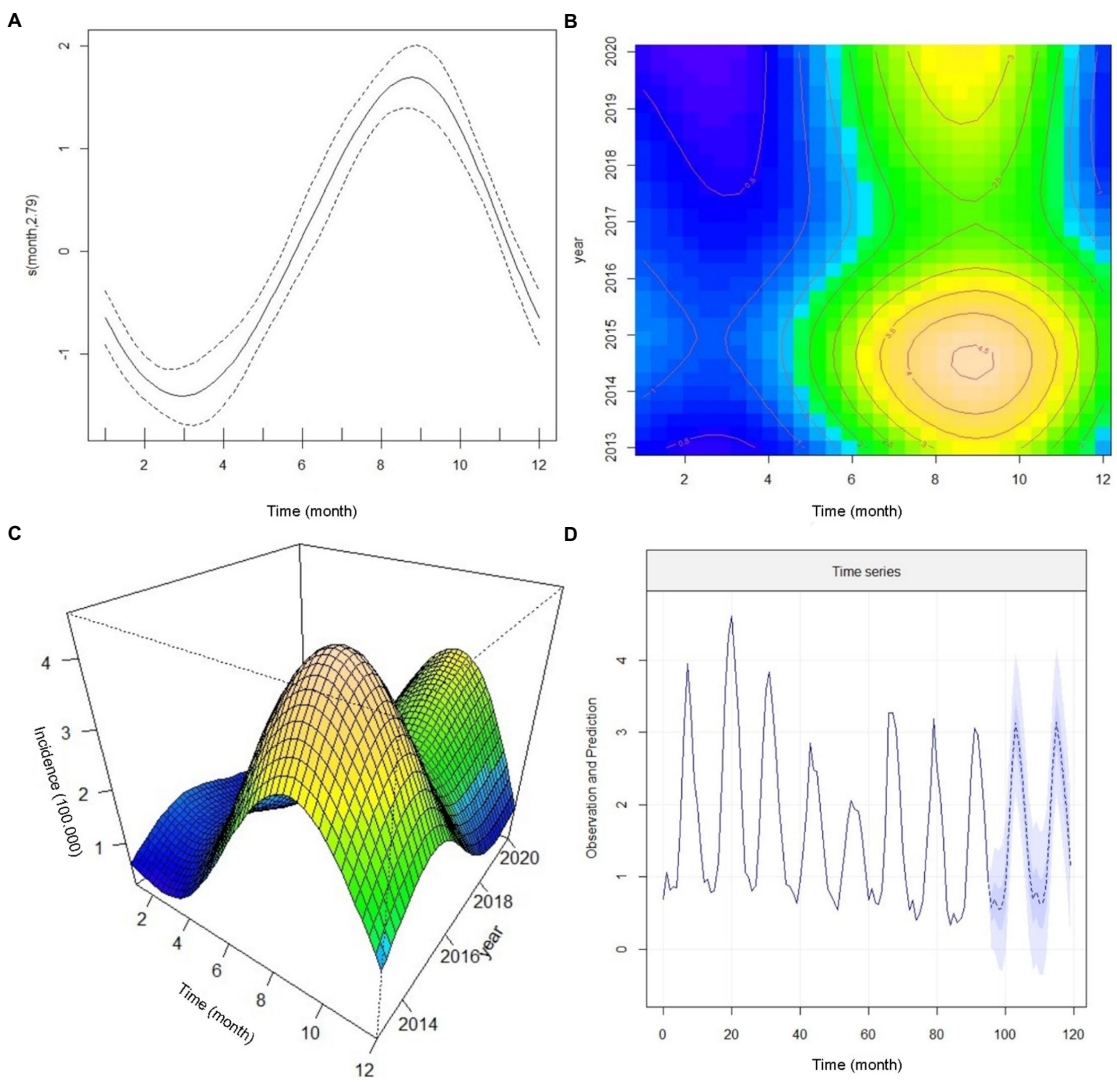
The annual incidence trend of cutaneous leishmaniasis in various provinces of Iran by the generalized additive mixed model (GAMM), 2013–2020. **(A)** Confirmation of a non-linearity model of incidence trends **(B)** a Contour analysis-based seasonal cycle displaying the incidence trends, **(C)** A 3-dimensional monthly-annual image of the incidence trends, and **(D)** Predicting the trend in the number of new cutaneous leishmaniasis cases from 2013 to 2022.

Next, we closely evaluated the incidence trends by the year of occurrence. We found that the highest number of reported morbidities appeared in 2014 and 2015 (Figure 2C). It was interesting to know the nature of non-linearity trends of cases in different provinces during the study. We found a robust non-linearity model analyzed by the GAMM model as the actual degrees of freedom (EDF) was 2.79 (*p* < 0.00 l) (Table 4). Further, we wanted to be sure of the non-linearity of the incidence trend; the outcome confirmed a similar distribution pattern of a non-linearity model (Figure 3A).

**Table 4:** The generalized additive mixed model (GAMM) represents the strong non-linearity of cutaneous leishmaniasis in different provinces of Iran, 2013–2020.

Smooth term	EDF	RDF	*F*	Value of *p*
S (months)	2.79	3	57.51	<0.001

EDF, Effective degrees of freedom; RDF, Reference degrees of freedom; < 1 linear, 1–2 weak non-linear, and > 2 strong non-linear.

Figure 3B illustrates a contour analysis image based on the monthly and seasonal cycle of CL incidence by the time of occurrence. We found that in early spring, the incidence rate was static (blue color), and in early summer (July), the patients rapidly increased where they reached a peak in December (brown color) and decreased after that. This image also shows that the highest number of new cases appeared in 2014 and 2015. Considering the yellow and brown colors in the upper part of Figure 3B, we expect a peak incidence of new CL cases from 2022 to 2023. The emergence of cases shifted from August to September in the current date and the cessation of cases is delayed from January to December. Figure 3C displays a 3-dimensional image of monthly and annual incidence peaks. One of the peaks occurred in 2014 and 2015, and the other is expected to occur in 2022 or 2023. In addition, this image (Figure 3C) shows a non-linear association of the new CL cases.

To forecast the incidence of CL in Iran, we used the Seasonal Autoregressive Integrated Moving Average (SARIMA) model. This model can be a valuable tool to predict the trend of CL in planning future public health programs. Therefore, based on the previous data, we developed a SARIMA time series model from 2013 to 2020 to predict the monthly incidence of CL for 2021 and 2022. Cutaneous leishmaniasis incidence in a given month can be assessed by the number of cases occurring in 2021 and 2022. The prediction data illustrates a non-linear seasonal incidence trend similar to 2020 (Figure 3D). More supplementary data to confirm the prediction assessment are shown in Supplementary Figure 1. We presented the trend values, including months, and point forecast at 95% confidence interval (CI) (Table 5) to closely evaluate the incidence. During September and March 2022, the incidence rate coinciding with autumn and winter (cold months) was statistically significant while not substantial in other months of the year. The plan requires a deep commitment to initiate interventional measures, implement appropriate control strategies, and highlight temporal and spatial hotspots. Identification of the persistent and high-incidence areas could help the program prepare and initiate an elimination plan for future control programs, as WHO requested (58).

**Table 5:** Prediction of cutaneous leishmaniasis incidence based on 95% CI for point forecast, 2022.

The month of 2022	Point forecast	%95 CI for point forecast
Apr	0.33	(−0.84, 1.50)
May	0.50	(−0.67, 1.66)
Jun	0.37	(−0.79, 1.54)
Jul	0.42	(−0.74, 1.59)
Aug	0.59	(−0.58, 1.75)
Sep	1.50	(0.33, 2.67)
Oct	2.48	(1.31, 3.65)
Nov	3.05	(1.89, 4.22)
Dec	2.95	(1.79, 4.12)
Jan	2.44	(1.28, 3.61)
Feb	1.85	(0.68, 3.02)
Mar	0.98	(−0.18, 2.15)

CI: Confidence intervals are zero non-significant, while the positive values are significant.

Furthermore, in the present study, we utilized the R 4.1.1 software and spatstat, shapefiles, maptools, rgdal, tmap, raster, sp., and spdep packages to identify hot and cold spots in the data, as well as spatial outliers. We used both the Global and Local Moran’s I to demonstrate the dimension of spatial autocorrelation statistics to detect clusters or local outliers, understand their contribution, and provide a decomposition to the Moran’s I global clustering statistic. The spatial Autocorrelation (Global Moran’s I) tool simultaneously measures spatial autocorrelation based on feature locations and feature values. The tool calculated Moran’s Index value and both a Z-score and *p*-value to evaluate the significance of that index.

Tabular numbers and maps illustrate that high or low values tend to concentrate in some provinces more than others. These geographical patterns reflect variability in the socioeconomic and environments of the studied population and species differences of the causative *Leishmania* parasite and the host’s immune response. We specifically examined the nature of the sample to distinguish the “fitted (smooth) from the “residuals” to follow descriptive data analysis terminology. This correlation may be different as low, high, or non-existent based on the variables used. Therefore, four categories were illustrated based on Moran’s values related to the locations and surrounding provinces. We highlighted the connection between the Moran scatter plot and the cluster map. The Moran clustering map provides a classification of spatial association into four categories corresponding to the location of the points in the four quadrants of the plot. Locations with high values and similar neighbors are evaluated as high-high (“hotspot cluster”). Locations with low values and similar neighbors are referred to as low-low (“cold spot cluster”). Low-high locations have low-risk but high-value neighbors (potential geographical outliers or “cold outlier areas”). A high-low (“hot outlier area”) is a region with high values surrounded by provinces with low risk. The local Moran statistic is constructed from the average incidence of the neighbors, which is sensitive to the effect of outliers.

**Figure 4:**
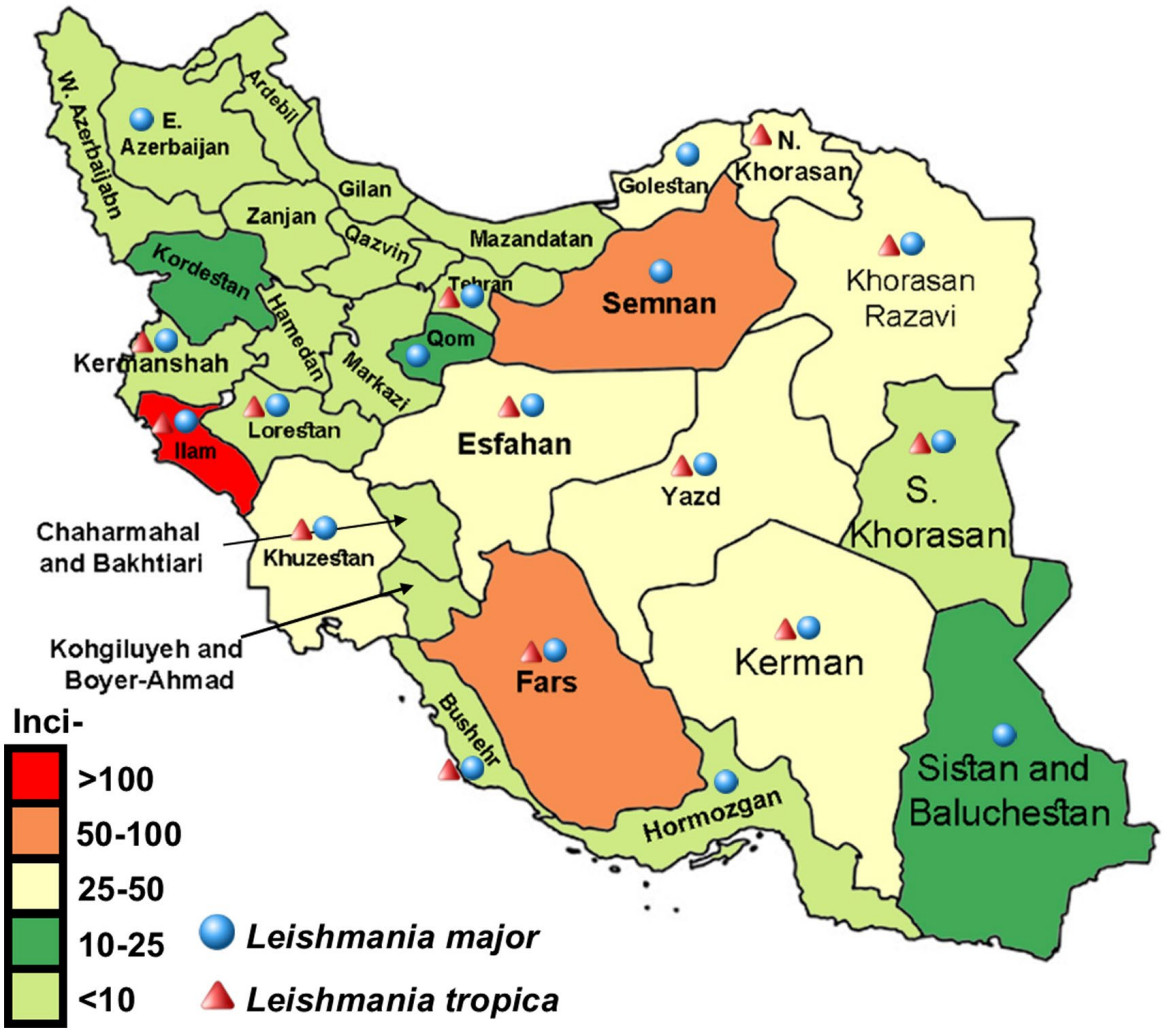
Displaying distribution and average incidence rate (2013–2020) of zoonotic cutaneous leishmaniasis and anthroponotic CL caused by *Leishmania major* and *L. tropica,* respectively. Infections with both diseases are detected in most provinces of Iran.

**Figure 5:**
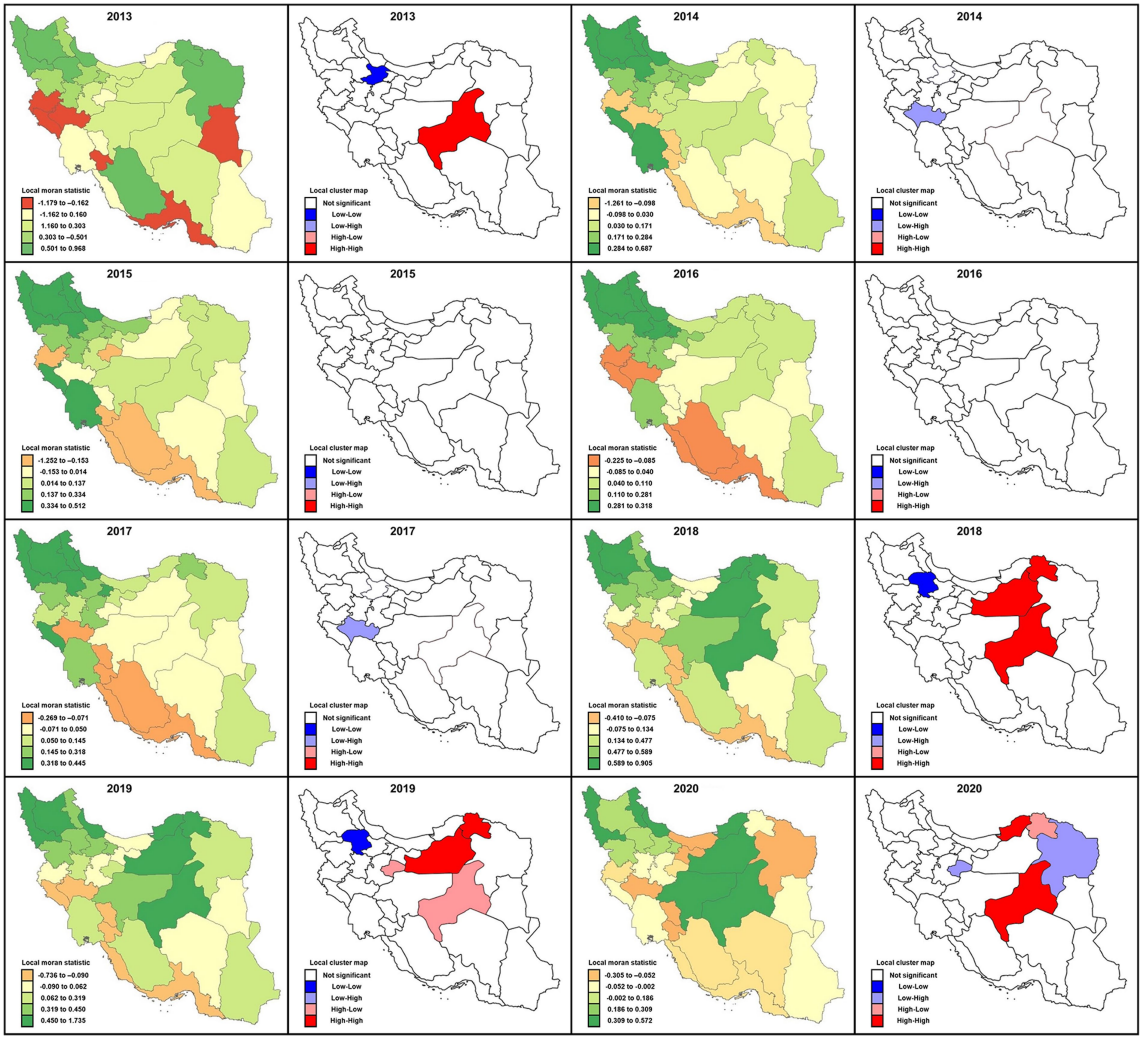
Local Moran’s I statistics and local cluster map for spatial autocorrelations illustrating clustering locations of cutaneous leishmaniasis distribution in different provinces of Iran, 2013–2020. Moran’s value has been mapped to highlight the incidence rates according to relative importance and surrounding neighbors’ behavioral relationships possessing four different categories: high-high, low-low, low-high, and high-low.

These groupings can directly interpret provincial incidence over time in the nationwide areas. Whole data were used to construct maps to interpret disease behavior and endemic provinces of the country and demonstrate the temporal expansion of disease incidence over the years and geographical areas. In the following maps (Figures 4, 5), green, yellow, brown, and red reflect Moran’s I incidence rates representing a specific topographical pattern. As the trends shift to darker green, the spatial autocorrelation becomes positive (high-high or low-low cluster). While the colors turn to brown or red, the status of provinces shows high-low or low-high. For example, in 2013, the spatial autocorrelation demonstrated a high cluster; the incidence in Yazd province was high, and the value was also significantly high in those spatially autocorrelated provinces. In contrast, Zanjan province revealed a low-low situation simultaneously, and the incidence rate was significantly low in neighboring provinces.

In 2017 like in 2014, the spatial autocorrelation signified a low-high outlier value in the cluster map for Lorestan province. As a result, the average incidence of the neighbors turned out to be much higher than would be the case under spatial randomness. Conversely, in 2018 the situation was similar to 2013; that is, the spatial autocorrelation presented low-low cluster locations in the map exhibiting similar clusters of low incidence rates in the adjacent provinces. Also, in 2018, several provinces, including Yazd, Semnan, and Khorasan Shomali, characterized high-high cluster locations confirming both high incidence rate cluster centers (with blue outlines) as well as their neighbors. The spatial autocorrelation picture was substantially different in 2019 relative to 2018. The status was the same for Semnan, Khorasan Shomali, and Zanjan; (low-low), while high-low for Yazd and Qom provinces, and the incidence rates changed to a high-low value. Unlike in 2018, these latter provinces indicated high incidence rates surrounded by low-value neighbors. In 2020 the circumstance was noticeably mixed. Yazd and Golestan provinces were high-high, but Qom and Khorasan Razavi were in high-low positions.

Based on the results of this study, the average incidence rate significantly reduced during 2013–2020, and the overall spatial autocorrelation was significant (*p* < 0.05). However, an exception was for 2015 and 2016, which showed no significant level (*p* = 0.06 and *p* = 0.07, respectively) when the average incidence rates were evaluated (Table 6). The highest spatial autocorrelation belonged to 2018 (Moran’s index = 0.25) and the least related to 2016 (Moran’s index = 0.08).

**Table 6:** Annual nationwide cutaneous leishmaniasis average incidence rates/100,000 and investigation of spatial autocorrelation using the Global Moran’I statistic, 2013–2020.

Year	Average incidence rate	Moran’s I statistic	Moran’s I standard deviate	Value of *p*
2013	19.60	0.17	1.93	0.02
2014	28.90	0.11	1.84	0.03
2015	25.10	0.10	1.55	0.06
2016	20.10	0.08	1.45	0.07
2017	16.40	0.11	1.82	0.03
2018	18.90	0.25	2.59	0.004
2019	17.10	0.21	2.38	0.008
2020	19.80	0.11	1.75	0.04

## 5. Prevention and control measures

Prevention and control of CL require an integrated effort entailing a combination of intervention strategies as transmission occurs in a complex biological-environmental scheme involving the parasite, host (human and/or animal), and vector. Control measures vary from region to region and are prioritized based on an allocated budget, although the approach mainly depends on costly, ineffective, and challenging chemotherapies. Key preventive and therapeutic measures are listed below:

### 5.1. Personal protection

Protection of the population by repellents such as diethyltoluamide (DEET), insecticide-impregnated nets (pyrethroids), windows, clothing, fabrics, curtains, and door fences was frequently used in different endemic foci within the country. However, their beneficial aspects have not been well evaluated in different localities (60). Dressing the skin lesions dramatically reduced contact between humans and sandflies (61).

### 5.2. Chemical control

The essential methods for controlling sandfly vectors with chemical insecticides are indoor residual spraying, spraying of resting sites of wild species, use of insecticide-impregnated resources including bednets and curtains, and pyrethroid-impregnated dog collars. Data indicated that insecticide-impregnated bed nets, indoor residual spraying with insecticide (IRS), and long-lasting insecticide-treated nets (LLITNs) were highly effective against ZCL and ACL primary vectors; however, they are not well accepted behaviorally by the residents (61, 62).

### 5.3. Environmental modification

Management of the environmental alterations has had a drastic effect on the frequency of vectors, resulting in a reduction in phlebotomine-human contact, or sandfly population, and transmission levels. This strategy may include the replacement of inhabitants away from sandfly habitations and physical modification of their environments. Elimination of propagating multiple risk factors such as solid wastes, garbage, maneuvers, leaves, organic remnants, and rubbles had a crucial role in peri-urban and high-risk areas in controlling vector densities and transmission profiles (25, 63). The destruction of the habitat followed by the exploitation of the land by settlements and farming is the only permanent way of controlling sandflies. This approach is successful but at a great expense in a highly endemic ZCL focus transmitted by *Ph. papatasi* in central Iran (64).

### 5.4. Gerbils and dogs’ control

Control of competent reservoir hosts is fundamental in ZCL and ACL, as control strategies can be directed at these hosts. There are numerous experiences gained by investigators in central Iran, where zinc phosphide poison mixed with wheat grains and vegetable oil (2.5%) were used in a radius of 500 m from houses, once a month during May, June, July, and September in the first year and repeated once every two years in the coming years for ZCL (49, 65). They suggested that gerbil control actions employing zinc phosphide within a 500-meter radius of houses once every two years in late April before the commencement of the active season of sandflies. In foci where dogs are reservoir hosts of *L. infantum* and probable hosts of *L. tropica,* the control of all dogs by sheltering, as practiced in a few places in Iran, is an efficient complementary means of ZVL and ACL control (66–68). As implemented in some localities in Iran and abroad, the selected culling of seropositive dogs was followed by a drop in canine infection while it is costly (69). A cheaper and more likely economic means of fitting dogs with deltamethrin-impregnated collars act as a long-term depot releasing the insecticide into the skin’s lipid (64).

In Iran, guard dogs were protected against 80% of bites of *Ph. papatasi* eight days after collars were attached (70). However, the effectiveness of dog collars will depend on the importance of domestic and wild canids as reservoir hosts of *L. infantum* or probable secondary hosts of *L. tropica.*

In areas where *L. major* induces CL, reducing rodent populations by physical destruction of burrows, followed by planting, has proved to be an effective and sustainable method of controlling CL where *Psammomys obesus* is the known reservoir. Poisoning with wheat grains and vegetable oil is effective for other migrant rodents such as *Meriones* (71).

### 5.5. Health education

Training programs for health care providers on various aspects of leishmaniasis, including rapid detection, case management, different control modalities, and rapid response to epidemics, have continuously been carried out in provinces where CL control units or centers were functioning as Kerman, Isfahan, and Bam. Education is undoubtedly a very cost-effective preventive measure (72–74). Notifying populations in endemic areas leads to appropriate case detection, a better acceptance of preventive and therapeutic measures, and lower behavioral risk. Fortunately, significant systematic health education has been implemented to evaluate different aspects of effectiveness and health impacts at the district and provincial levels. A cost-effectiveness education in Iran and elsewhere assessed the advantage of combined prevention strategies for CL-endemic areas, including applying insecticide-impregnated clothing and curtains plus premature CL diagnosis training plans for health care personnel (75, 76).

### 5.6. Treatment of patients

Based on the WHO guidelines, cases of ZCL do not need to be treated unless vital organs are affected. In contrast, patients with ACL must be treated since humans are the only reservoir host (77, 78). The type of treatment is based on five criteria (79), including the size of the most extensive lesion, the number of lesions, the location of lesions, the causative agent (type of *Leishmania* species), and in some instances, immunologic status. Meglumine antimoniate (Glucantime®) is the drug of choice, administered intralesionally once a week for 8–12 weeks along with biweekly cryotherapy or intramuscular injection alone for 3–4 weeks according to the national guideline consistent with that of WHO (20, 79). Another second-line medication, mainly in combination with MA, was used on a special occasion. The overall efficacy of MA against ACL was 89% in a cohort study with a significant focus in southeastern Iran (51); however, in routine practice, variable ranges of treatment failures have been reported for ACL (80–82) and ZCL throughout the country (82, 83). In a contraindication for conventional therapy of CL, other treatment choices, particularly multiple therapies, were used (78).

## 6. Major challenges

### 6.1. Associated-risk determinants

Cutaneous leishmaniasis is linked with numerous confounding factors. The following risks are the significant determinants associated with the perpetuation of the organism, which is directly involved with the outbreaks caused by ZCL or ACL in Iran.

### 6.2. Agricultural developments

Launching agricultural projects has increased the risk of ZCL in rural areas. Under such conditions, anthropogenic ecological disturbances occur, and simultaneously, many non-immune immigrants intrude into the area where the sylvatic ZCL cycle is present (84, 85). Transmission to humans is promoted by people sleeping outdoors without bed nets throughout the transmission months. There are many examples of these epidemics where new agricultural developments in Iran (86, 87). One example was the epidemic of ZCL in the southern villages of Baft district with high severity in all age and sex groups in a new agricultural region.

### 6.3. Socioeconomic factors

Primitive and poor housing conditions and the resulting low living standards increase the risk of transmission in peri-domestic areas. These are organic remnants, manures, piles of bricks, debris, rotten leaves, and stones, which constitute potential breeding areas and resting sites for sandflies. Such places in the vicinity of poor households, and low-income individuals such as laborers, favor CL transmission. Also, many people sleeping in the shared room attract peri-domestic anthropophilic sandflies; therefore, poverty increases the disease risk. Housing sanitary conditions, including the lack of solid waste management and sewage treatment plants, could provide vectors’ resting sites and enhance sandflies’ proliferation potential, facilitating contact with humans. Sandflies are frequently attracted to crowded housing, which provides a good source of blood meals. The human behavioral attitude of sleeping outside and on the ground could also increase the risk of infection. Such risk-associated determinants are frequently observed as the cause of epidemics/outbreaks in Iran (88–90).

### 6.4. Climate changes

Leishmaniasis is a highly climate-sensitive disease, and epidemiological characteristics can be changed in several ways: physical factors include temperature, humidity, precipitation fluctuations, influence vectors, and reservoir populations (91). Even slight fluctuations in temperature can substantially affect the biological development of the promastigote stage in the gut of sandflies contributing to the transmission of the organism in the previously non-endemic areas (18, 92). Drought, flood, starvation, and disaster contribute to enormous migration and population displacement to new endemic foci, creating an epidemic situation of variable magnitude. Such conditions provide new risk factors that favor the parasite, vector, and reservoir, leading to the disease’s propagation. Extreme examples have previously been documented in the CL epidemic foci following the presentation of risk factors in favor of illness (14).

### 6.5. Population movement

Outbreaks of CL are often linked with population displacement from non-endemic areas to new places where the CL infection is already present. The movement of inhabitants and the creation of new settlements in suburban areas in close vicinity with endemic foci are major confounding factors and are directly connected to the incidence of CL, either leading to exposure of the susceptible and at-risk population to new hazards or contributing to the introduction of the causative parasite into new areas. The estimate of such outbreaks is related to the accessibility of ecological and epidemiological information. After the Bam earthquake in 2003, approximately 60,000 newcomers arrived to provide services to the survivors. The city was divided into 12 zones, and all zones were given to health personnel from different provinces of Iran. A massive outbreak of ACL occurred two years after the earthquake and was sustained for nearly four years. In this outbreak, the number of cases increased from 800 to 5,000. In addition to providing numerous risk factors, the new arrival population was vulnerable to CL causing such a continued and enormous epidemic with variable proportions (14, 93).

The spread of ACL in southeastern Iran, particularly in Kerman province, has resulted from population movement and the provision of many risk factors for the proliferation of vectors and transmission of the disease (14). Several emerging epidemics of ACL occurred in rural areas within and outside the County of Bam including, Dahbakri, Nazamshar, Ghal-e-shahid, and Mohammadabad (51, 52, 55, 93–97). The epidemic of CL is frequently connected with the movement of non-immune people into foci where active transmission is already present. The epidemics of varying scales have been reported due to population movements for economic reasons and agricultural development in Iran and other countries such as Afghanistan, Brazil, Libya, Iraq, Sudan, Syria, Morocco, Peru, Saudi Arabia, and Yemen (1, 24, 98–104).

### 6.6. Environmental changes

Ecological changes in the landscape in rural and urban areas where the disease is endemic contribute to the outbreak. As a result, many risk factors favoring sandflies’ breeding potentials will be provided, leading to a sudden or gradual increase in cases. Garbage left around houses permits some localities to store grain outside houses attacks rodents, serving as the reservoir for the propagating disease. One of the reasons for the low number of CL in the past decades was the implementation of mass insecticide spraying in high-risk areas around human and livestock dwellings (105).

## 7. Other challenges and gaps

### 7.1. Health care system

In 1985, relevant health fields and medical education were initially combined within the Ministry of Health and later on in 1993, renamed MOHME. Therefore, both sections, medical education, and health services were integrated and formed Universities of Medical Sciences and Health Services throughout the country’s provinces. At present, MOHME is in charge of both tasks; providing health care services for the communities and training human resources (106, 107).

Although some posts may be subject to change over time. The healthcare system in Iran is based on three pillars including the public-governmental system, which provides most services, the private sector, and NGOs.

There are numerous health system barriers potentially affecting various segments of control programs. The government and the patients, including the heavy economic burden, sometimes logistic problems in accessing primary healthcare centers, and treatment costs, although offered free, contribute to poor adherence to treatment. Since the burden of CL has diverse dimensions, this requires sustained and tremendous efforts and actions from the government such as policy-makers and healthcare providers, private sectors, and national and international agencies, notably WHO. Other health system challenges entail insufficient capacity for surveillance, especially at the peripheral level. The number of health facilities is not adequate in some areas, health personnel often have scant experience, supplies are not adequate, and the procurement supply chain ensures that medicines available at request are not sometimes enough. Lack of sufficient budget and inter-sectoral coordination, fast turnover of health personnel, lack of district and provincial attention to control strategies, and many other related issues are often unsatisfactory.

### 7.2. Health services

Health services are provided at primary, secondary, and tertiary levels, including health houses, and health centers (urban and rural, hospitals at district and provincial levels). Most of the primary health needs are provided by PHCs in health houses, followed by higher professional personnel and physicians in rural and urban health centers. Hospitals are responsible for providing secondary health care services for the residents. Health expenditure has increased rapidly in the last decade, but there is still a shortage of healthcare financing. The out-of-pocket expenditure share used to be around 50%, although this ratio has significantly changed to a lower proportion during the past seven years, mainly provided by several public and non-public insurance schemes. Human resources have increased to approximately 300,000 employees, including health, clinical, and educational services at different levels. Leading health indicators have improved mainly over the past three decades, owing to the government’s strong commitment to the effective delivery of PHC and inter-sectoral development (108, 109).

The decision-making body is centralized, directly influencing on-time decision-making, particularly in emergency events and responses. Decentralization of leishmaniasis control is planned, implemented, reported, monitored/evaluated locally, and supervised centrally by the health authorities at provincial and CDC members at the national levels. Retirement of staff and health personnel with no substitution retards the whole process of the control activities. Government is not entirely able to substitute retired personnel officially.

### 7.3. Poor follow-up assessments

There is inadequate follow-up examination of patients afflicted with CL following treatment regimens. There seem to be some patients having absent from the treatment follow-up assessment. Such underprivileged follow-up examinations contribute to treatment failure and intensification of cases, notably in ACL endemic areas. The primary barriers are as follows (51):

- Non-availability of medicine
- Long-distance to healthcare centers
- Remote areas of living conditions and scattered residents in the affected areas
- Receiving incomplete treatment adherence
- Work constraints

### 7.4. Suboptimal reporting

In addition to the insufficiencies above, which influence the control measures, there are also essential obstacles that could play a precipitating role in the optimal provision of healthcare services (110, 111).

The majority of official data are passive case detection, which is reported via the PHC surveillance system. Many cases are unreported, undiagnosed, or misdiagnosed, especially in remote areas and poor endemic villages where the availability and accessibility of diagnostic methods are scant (77, 112), while the actual burden might be worse, and the reports from the PHC surveillance system reveal only a fraction of the actual picture (7, 113).

### 7.5. Cross-border movements

Cross-border movement and tourism from neighboring endemic countries for ZCL and ACL are major challenges for a comprehensive control program. It is essential that some types of cooperation with the health authorities of these countries should be set up to combat, control, and settle such challenges threatening the program. Migration of non-immune populations from non-endemic areas to endemic regions and *vice-versa*, population movement, and migration from endemic areas to non-endemic localities where the conditions for propagating the parasite, vector, and reservoir are provided could gradually or promptly abrupt into epidemic conditions (52, 114, 115). Iran is connected to Turkey, Azerbaijan, Armenia to the northwest, Afghanistan, and Pakistan to the east, Turkmenistan to the north, and Iraq to the west. The country border around 499 km with Turkey, 432 km with Azerbaijan, 35 km with Armenia, 909 km with Pakistan, 936 km with Afghanistan, 992 km with Turkmenistan, and 1,599 km with Iraq. Iran borders 650 km also, along the Caspian Sea. The above boundaries are aside from 2,440 km coastline sea borders with six other Arabian countries, including Kuwait, Oman, Qatar, Bahrain, the United Arab Emirates, and Saudi Arabia.

### 7.6. Lack of side-benefit of previous anti-malarial activities

Although it is controversial, marked changes as a side benefit of anti-malarial activity and a reduction in the number of sandflies have previously been reported. Following the cessation of spraying houses and dwelling areas, a resurgence of CL cases was observed. Discontinuation of spraying actions against malaria could be a protective factor for leishmaniasis in malaria areas where the incidence of the disease has significantly increased (49, 116). Also, house spraying with insecticides to control malaria in the 1950s and 1960s was experienced by reducing the kala-azar (VL) in India caused by *L. donovani* and transmitted by *Ph. argentipes* (116).

### 7.7. Demographic transition and fast urbanization

There is one-third of the population between 15–30 years old. The vulnerability of the three age groups to social and behavioral problems makes them at risk of suffering from major health problems like opium and drug addiction, violence, traffic injuries, and psychological complications, contributing to economic burden and other financial crises (117). One of the most outstanding landscapes of recent decades has been the wild urbanization rate. The urbanization pattern and socioeconomic status can promote disease transmission concentration, contributing to injustice in allocating medical and healthcare facilities and creating hotspots of diseases within communities, towns, and cities for the rapid spread of emerging diseases (118).

In Iran and most developing countries, mass human migration has lately been directed to unpredicted enlargement challenges of diverse ‘megacities,’ where living standards for sanitary conditions are unsatisfactory. Hence, such situations create appropriate breeding environments and augment human contact for spreading vector-borne diseases (VBDs), such as leishmaniasis (119, 120).

### 7.8. Cutaneous leishmaniasis outbreaks

In recent years Iran has experienced several outbreaks of CL due to natural events, man-made environmental alterations such as fast urbanization, deforestation, and population displacement for economic reasons, including agro-industrial projects, road constructions, and widespread migration more frequently from rural to urban areas in small towns and large cities (e.g., current droughts and earthquakes) (55, 56, 93, 94, 97, 121).

### 7.9. Natural events

Iran is highly vulnerable to natural and man-made (anthropogenic) disasters, particularly earthquakes, flash floods, and droughts (14, 86). Iran is ranked first regarding the frequency of earthquakes *per annum*, with approximately 5.0 to 7.0 on the Richter scale. This country is also ranked first in relative vulnerability and survivors each year due to earthquakes. Approximately 75% of the country’s major cities are located in potential earthquake zones. Seasonal flash floods have drastically increased, whereby many local people in many provinces died, and their houses, fields, and livestock were devastated in May 2019. In Iran, it is estimated that 5 to 10% of the annual GDP budget has been allocated to manage disasters (122). Following disasters so many risk factors in favor of the propagation of vectors and multiplication of reservoirs will be created, and as a result, epidemics of varying degrees of CL will occur (96, 123–125).

### 7.10. Clinical forms, diagnosis, and treatment

Clinical assessment of CL lesion combined with identifying its causative *Leishmania* agents is crucial for selecting proper therapeutic modality and designing appropriate strategic approaches, especially in ACL endemic foci caused by *L. tropica* where control measures are limited to early case detection and prompt treatment of patients. Since CL lesions, acute or chronic mimic a variety of disease conditions, notably a broad range of viral (zoster, herpes-like, and wart viruses), fungal (lupus vulgaris, and sporotrichosis), bacterial infections (tuberculosis, and mycobacterial ulcers), skin diseases, tropical ulcers, myiasis, acute furunculosis, ecthyma, foreign-body granuloma, sarcoidosis as well as some parasitic infections and cancers (carcinoma of the skin); knowledge of such disease presentations and confirmation of the complication based on demonstration of the parasite is essential for any dermatologic practice in endemic areas (51, 77, 78, 126, 127).

Both CLs; ZCL, and ACL, generate a broad spectrum of disease manifestations in Iran and abroad. In addition to “dry type” and “wet type” lesions, typically represented by ACL and ZCL, respectively, there are also several atypical clinical forms that are presented depending on the condition; the causative *Leishmania* species or variant and state of the host’s immune response, typical stages of skin lesion including papule, plaque, ulcerated nodule, and ulcerated plaque (52).

### 7.11. Drug unresponsiveness

Leishmaniasis is treated with pentavalent antimonial agents such as MA. Meglumine antimoniate (Glucantime®) and sodium stibogluconate (Pentostam®) have been a mainstay of treatment in the past 80 years, but resistance to these drugs has significantly increased in the endemic foci throughout the provinces of Iran and worldwide as well (78, 128). Failure is a frequent phenomenon reported for many years in treating CL in Iran and around the globe. Antimonial compounds have been used for many years in Iran and many countries despite their parenteral administration, toxicity, resistance, and long duration of applications. There are reasons for the emergence of resistance in Iran. Several determinants have facilitated this phenomenon, including the high level of endemicity of the *Leishmania* species, the high proportion of routinely treated patients, and the most exclusive human-to-human transmission. Various reports have demonstrated such treatment failure in several localities within the provinces (12, 51, 129, 130).

Meglumine antimonite has extensively been used against all forms of leishmaniasis in Iran. Variable ranges of treatment failure against CL have previously been reported from endemic areas; thereby, many patients with ACL have developed unresponsiveness to Glucantime®; the rate of clinical failure of ACL patients in experimental models was reported to be approximately 12 to 15% in Mashhad, Kerman, and Bam (130, 131). *L. tropica* causing ACL is restricted to humans as anthroponotic species drug-refractory is a significant challenge in properly treating CL cases. Humans are the sole source of reservoir infection. The patient should be treated to prevent further dissemination of the organism to sandflies and, in turn, susceptible individuals. Also, some drug failures against ZCL have been reported from endemic foci since the disease undertakes an acute course and humans are incidental hosts. Presumably, non-treated cases have no role in transmitting the disease; although, the patients are often treated with proper compounds, accordingly. Suboptimal doses of the drug and poor treatment adherence could be predisposing factors for developing drug unresponsiveness (50, 81, 130, 132, 133).

Control of ACL caused by *L. tropica* is primarily based on early detection of the CL cases, diagnosis, identification of the causative agent, and prompt treatment via an effective surveillance system (51). Given that humans are the only reservoir host, untreated chronic cases such as leishmaniasis recidivans (lupoid leishmaniasis) remain the infective reservoir for disseminating the organism (95, 134). In general, treatment failure has been somewhat neglected in the conventional delivery of PHC services due to non-compliance with treatment protocol by patients. A robust assurance of a multidisciplinary method is necessary to make advancements in this part. Therefore, coordinated action from health experts, investigators, health providers, and strategy–makers is required. Treatment consequence is a multifactorial issue determined by the interaction of multiple factors (50, 51, 88, 135, 136).

Poor adherence to the treatment occurs for various reasons, including doubt about the expected benefits, the efficacy of treatment, unpleasant side effects, work constraints or economic situations, traveling away from home, feeling sick or depressed, and simple forgetfulness. In the evaluation of unresponsive cases, a considerable number of patients show difficulty in adhering to their recommended therapeutic regimens. The facts above represent Glucantime® mismanagement as a critical contributor to failure in the country’s endemic areas. Treatment failure should be monitored to maintain the life span of existing antileishmanial drugs, delivery, and clinical response. Healthcare providers should pay special attention to CL patients who receive partial treatment regimens and closely monitor such patients to reduce the chance of drug failures (111, 137–139).

### 7.12. Vaccines development

Vaccination against CL has been used in humans for over 70 years. In protozoal diseases of the world, inoculating infectious organisms from the infection of a sore in naked parts of the body is an antique exercise. Iran has taken the lead in vaccine development for leishmaniasis since the 1960s. *Leishmania major* skin test antigen (LST) was first produced by Alimohammadian and colleagues at Pasteur Institute of Iran (140), and an experimental killed autoclaved *L. major* (ALM) vaccine (141) by Dr. Fesharaki in Razi Institute (Karaj, Iran). The products have been given to different countries for conducting vaccine trials against CL or VL (142). Trials of single and multiple doses of the first-generation vaccine have been used in Isfahan and Kerman and supported by TDR/WHO and MOHME in Iran (95, 143–146). At present, there is no efficacious human vaccine available against any form of leishmaniasis (147).

The only available non-approved vaccine which can be used in certain circumstances is a live virulent strain of *Leishmania major*. Leishmanization inoculates live *Leishmania major* metacyclic promastigotes from a purified culture medium to produce a self-healing lesion. It was an occasional practice against CL in Middle East countries, mainly used in Iran, Uzbekistan, and Israel to protect against future lesion development (148). Historically, CL was found to produce lifelong immunity to reinfection. Moreover, only a tiny proportion of individuals infected with *Leishmania* agents develop full clinical symptoms of the disease, while most are either asymptomatic or self-curing. Leishmanization programs were initiated in a hyperendemic area (Isfahan), and a high-risk group participated in the Iran-Iraq war, where a massive epidemic of ZCL was sustained. Over two million people undertook leishmanization, and it was reported to reduce the incidence of ZCL significantly; however, it induces lesions lasting several months or years (149). The biggest problem was the development of non-healing forms at the site of inoculation (1%–3%). However, with the onset of HIV epidemics and low production quality, leishmanization has been abandoned, but it remains an option when people are at high risk of afflicting CL (150).

Currently, vaccine development against humans and even canines is under critical investigation in Iran and globally. Although some vaccines have been developed for vaccination against the disease in murine and canine models, in reality, they could not help eliminate leishmaniasis in either case (humans and dogs). During the past three decades, tremendous efforts and significant studies have been carried out in vaccine development, notably in Iran. However, due to the complexity of adaptive and innate immunity, the search for a vaccine against leishmaniasis gains an extensive opportunity to give rise to a long list of potential vaccine candidates against leishmaniasis. This also remains unsolved until more profound knowledge and tools become available.

The main challenges and limitations for the progress of an efficacious vaccine against various forms of leishmaniasis are briefly listed as follows (151):

- One of the main challenges in vaccine development against leishmaniasis is the poorly understood mode of host–parasite interaction and the complexity of the immune response associated with *Leishmania* species.
- Another main challenge is the proper immunobiological requirements for vaccine development against all types of leishmaniasis (CL, MCL, and VL).
- Lack of understanding of the many components that might cause such reactions.
- Establish trustworthy methodologies and approaches for assessing vaccination effectiveness.
- Develop acceptable animal models for preclinical vaccination effectiveness testing.
- Discovery of suitable adjuvants and delivery systems to elicit a protective immune response.
- High cost of vaccine production.

Finally, long-term memory cells are generated for protection.

## 8. Health facilities situation

### 8.1. Health system

In 1985, relevant health fields and medical education were initially combined within the Ministry of Health and later on in 1993, renamed MOHME. Therefore, both sections, medical education, and health services were integrated and formed Universities of Medical Sciences and Health Services throughout the country’s provinces. At present, MOHME is in charge of both tasks; providing health care services for the communities and training human resources. The health care system in Iran is based on three pillars: The public-governmental system, which provides most services, the private sector, and non-governmental organizations (NGOs).

## 9. Capacity and capability

### 9.1. Human resources

- There are various human resource development plans in various fields relevant to leishmaniasis, including physicians, laboratory technicians, environmental health and entomology bachelors, and masters of medical sciences. Most human resources should officially be the Iran Health Care Reform Plan initiated on May 5th, 2014. Such resources provide rural and urban health clinics, hospitals, and environmental health units for PHC and leishmaniasis management.
- In addition to their educational background, human resources gain skills through periodically planned programs during the recruitment period.
- There is a description of the job instructions to indicate their path, experiences, and skills, and there are opportunities for career progression at each health or clinical level. Their career pathways are also defined.

### 9.2. Personnel/staff/physicians at the national level

The nature of human resources is integrated, and the only exception is in the centers located in high endemic foci where the main tasks are restricted to leishmaniasis works. Presenting a precise list of human resources at various levels is impossible due to the vast variability of jobs and the integration nature of leishmaniasis in the country, where multiple CL foci in 60% of the country’s provinces are present.

### 9.3. Training

The personnel, staff, and physicians have to be trained periodically in health and related units, but it is not complete, and there are gaps in their training careers. The training system for new personnel in health units is located at the district level, where educational and training services are available. Professional health forces residents are trained in Bahvarzi schools, where these people learn professional units relevant to PHC. Other training experiences and skills are mediated through health managers or different faculties of the related universities.

### 9.4. Financial resources

- According to the CDC authorities, an annual budget is allocated for leishmaniasis control at various levels. The amount that the government is allocated for different activities such as planning,
- Implementation, surveillance, monitoring, evaluation, and reporting are not adequate to carry out routine tasks, but depending on the province, the expenses are variable.
- The budget for leishmaniasis is negligible and is not adequate for most activities as this disease is neglected. Therefore, there are major constraints and gaps in providing duties, particularly at a peripheral level where an at-risk population is present. The area in which the budget is allocated include:
- Disease surveillance, case detection and management, program management, reservoir host control, integrated-vector control, capacity building, and health education.
- It is of note that the PHC surveillance system, as previously mentioned, has its budget administered by the University of Medical Sciences (UMS) in the country, and they have their budget both for salaries and plans. For leishmaniasis, a sum of 10,400,000,000 Rials was initially allocated through CDC in 2020; but for some reason, nearly one-third of the budget was not allocated. Hence, the budget is not adequate to cover various management chains and interventions.
- Furthermore, there are still other sources of budget allocation where the authorities in UMS, sometimes through the governorate budget from public sources or proprietary revenues, shift the money for specific tasks. Nevertheless, the amount precisely allocated for leishmaniasis activities due to the complex nature of health issues is not well known.
- According to an inquiry from a responsible authority, the amount of money allocated annually for leishmaniasis activities is roughly around 30%–40% of the expenses required to manage the program.
- There are no other sources of funds to perform the tasks, and also there is a lack of global NGOs in support of plans for leishmaniasis activities.
- The post-elimination budget is another problem that the units are faced. It is supposed that the personnel should be substituted, annually regularly while it never occurs and therefore a significant amount of such deficient budget has been reduced.
- Approximately 10%–12% of CL patients are referred to higher hospitals or higher health facility levels for advanced therapy. Although these cases receive thoughtful attention and proper treatment modality, they often remain untreated, and the cases become chronic or transform into resistant clinical forms, which is one of the main problems notably in ACL foci where *L. tropica* is predominant.

### 9.5. Intra-sectoral collaboration

There is a reasonably good collaboration between various health sections within the MOHME. This is more likely prominent when a public health campaign at national, provincial, and district levels is conducted in the health system plans such as polio or malaria. Although leishmaniasis is generally well-focused and defined at any level, intra-sectoral collaboration needs more support.

### 9.6. Inter-sectoral collaboration

Since most health issues extend beyond the provision of health care services, a harmonized inter-sectoral collaboration between ministries, different governmental levels, NGOs, and stakeholders is crucial to address health issues and guide leishmaniasis actions at various levels. Basically, besides the University of Medical Sciences authorities, the health council at the provincial level strengthens and promotes such inter-sectoral cooperation, and coordination between all organizations within the ministry of health needs further attention. The council also works at the district level between the health authorities, the governor, and the representative of different administrations. Since the nature of health issues are multifactorial and multidimensional, the efficiency of such collaboration is variable.

### 9.7. Engaging and mobilizing communities

There is a national plan for community mobilization and participation in various health activities coordinated by the MOHME. As coordinated by health authorities and community engagement, many activities are carried out, especially in public education, awareness, and advocacy. There is a good experience with health volunteer forces participating actively in pubic-related health issues at different levels. However, since there is no defined monitoring and evaluation system, their effectiveness is not well apparent, although their works are well appreciated.

### 9.8. Surveillance system

At present, the most case detection system is based on passive case-finding approaches. This is why a substantial number of cases (2 to 5 times the detected cases) are not found (20). This under-reporting phenomenon further complicates detecting cases of ACL in endemic areas where humans serve as the primary source of infection and dissemination of the disease through the bite of the female *Ph. sergenti* at large in the area.

### 9.9. Case-management

In ZCL foci, since small gerbils are the primary reservoir hosts and the course of the disease is acute, there is no obligation to treat the patients unless a vital organ (e.g., face, nose, joints, and ears) is involved. However, early case detection, diagnosis, and prompt and effective treatment in ACL endemic areas are essential to reducing the principal source of infection (humans). Unfortunately, since most cases are passively detected, managing the diseases takes longer than 2–3 months. Therefore, this late treatment schedule contributes to drug unresponsiveness and leaves more complications and prominent scars. Many cases remain chronic, and some cases develop lupoid leishmaniasis, providing a source of infection for vulnerable populations in endemic areas. In addition to passive case finding, active case detection is highly recommended to decrease the disease burden. Improving public awareness and knowledge among vulnerable people to encourage early diagnosis and treatment-seeking behavior.

### 9.10. Basic and applied research agenda

Most basic and applied research needs and investigations, including the health research system, are centered in the UMS and research institutions. If there is a need, the above research bodies should be ordered and carried out. There is no agenda held for basic and applied research, but sometimes depending upon the priority of the needs, a research agenda will be established at the district or provincial level.

A search in Scopus reveals over 1,231 (approximately 317 articles on treatment and171 articles on epidemiology) articles of various types have been published from 2014 to 2020, and a PubMed search returns 1981 articles (including 925 articles about the treatment and 438 articles about the epidemiology of leishmaniasis) published during the same period. Most of such research is in epidemiology, treatment, and to a lesser degree, vaccine, and drug developments.

As leishmaniasis is a common disease in the country, almost all the UMS, Medical Schools, Faculty of Health, and Paramedical Schools are involved in research activities. The exceptions are leishmaniasis research centers and institutions such as the Dermatology Research Center in Isfahan, Tehran Center for Research and Training in Skin Diseases and Leprosy, Kerman Leishmaniasis Research Center, Pasteur Institute and the School of Public Health (and Parasitology Research Center), Tehran University of Medical Sciences, which are directly involved, and therefore, are responsible for most publications in the field.

Whether the research findings are reviewed, utilized, or implemented by the program is not well known, but the CDC is involved in applied research and vaccine development. Examples of this involvement are the Ampholeish project (liposomal formulation of amphotericin B) and the development of an attenuated *L. major* vaccine candidate is directly supported and assisted by the CDC.

### 9.11. Preventive measures

Since there are no efficacious vaccines available, prophylactic measures should be used systematically in endemic areas, but at present, these measures are used sporadically and only in a selective manner. ITNs and LLITNs are not behaviorally well accepted (20). Most people in endemic areas sleep late at night they become infected by the bite of female sandflies early at night. The screens are not affordable, and the local villagers do not use them at peripheral levels. Furthermore, most people avoid using impregnated curtains and clothing (62). Due to budget restrictions, there is no regular use of insecticide in endemic areas. Previously, insecticide spraying was used two times *per annum* in malarious areas, but currently, the number of malaria cases (incidence) in different rounds of elimination programs, as supported partially by WHO, has decreased to less than 200 cases. As a result, the cases have become limited to remote and scattered areas where spraying has most likely not been practiced. Therefore, the exact frequency of insecticide resistance is not well-known. Some findings showed that *Ph. papatasi* was resistant to DDT, deltamethrin, permethrin, bendiocarb, and susceptible to cyfluthrin. On the other hand, the *Ph. sergenti* collected from indoor and outdoor sites were susceptible to all insecticides (152, 153).

### 9.12. Data system

At present, portable data collection and reporting systems are imperative in endemic areas. Data are reported through districts to provincial levels and then to national headquarters (CDC).

### 9.13. Monitoring and evaluation

Monitoring is the routine follow-up examination of the leishmaniasis program, including data collection, analysis, recording, reporting, documentation, development of actions planning, and evaluation of public health practice. The main objective of monitoring is to evaluate progress and implementation status, detect challenges and limitations, and ensure accountability and decision-making based on evidence. Generally, the used indicators measure follow-up, processes, outcomes, or impact.

The monitoring of the leishmaniasis control plan is based on the list of indicators used to evaluate the program’s performance. Such indicators will provide evidence-based information on different phases of the control program at various levels: village, district, province, and national. All indicators, including epidemiological and operational indicators, are expected to be monitored and evaluated during the action plan.

## 10. Present status of the strategic position of leishmaniasis

### 10.1. Internal and external environmental analysis

**Figure 6:**
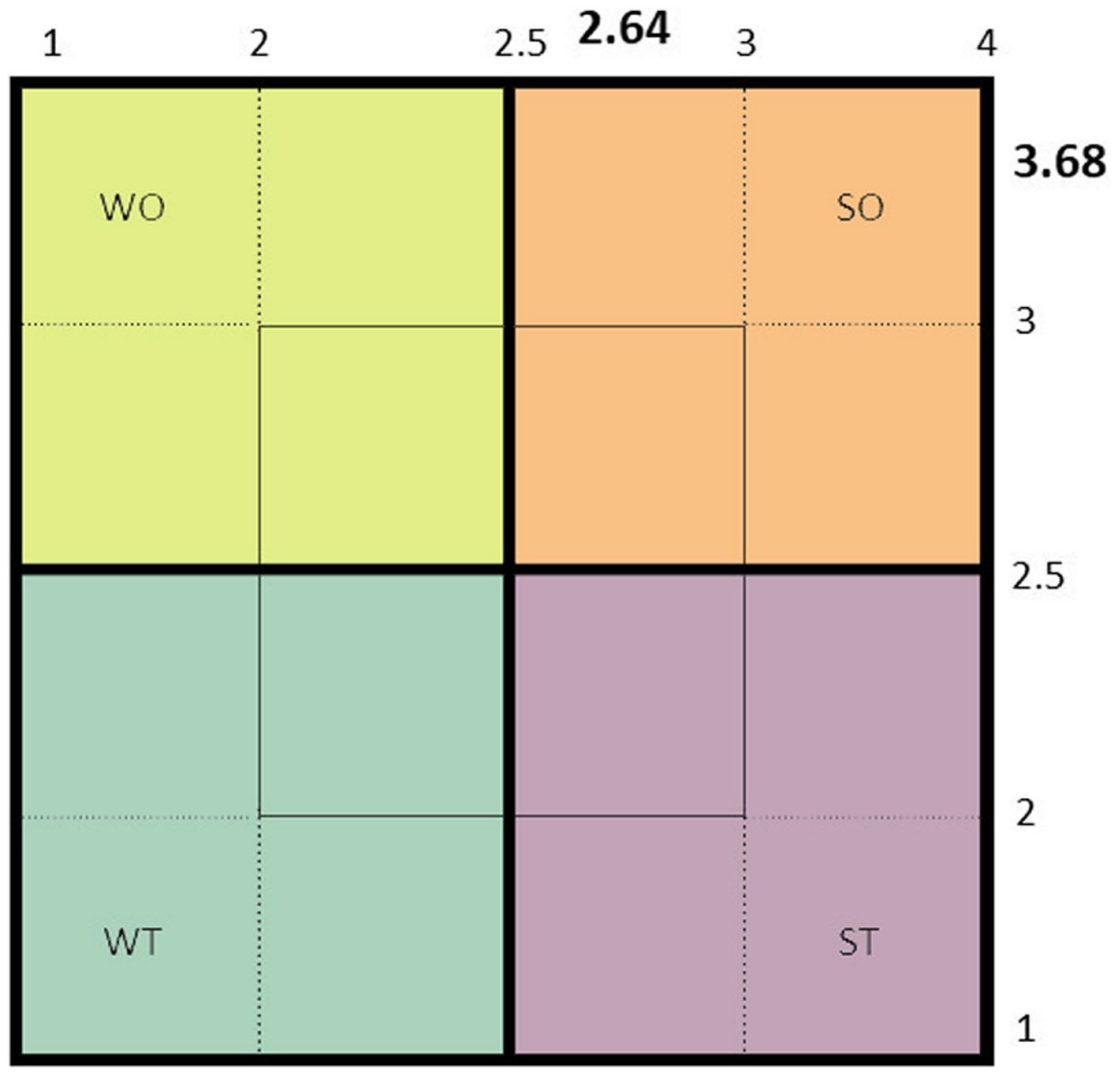
The present status of the strategic position of cutaneous leishmaniasis is based on the scoring scale of the strength, weakness, opportunities, and threats analysis (SWOT matrix).

A leishmaniasis expert team for assessing the gap analysis was established to find the leishmaniasis control program’s strengths, weaknesses, opportunities, and threats (SWOT) toward an elimination plan. The SWOT analysis was intended to facilitate a data-driven look and fact-based at the ongoing position of the leishmaniasis organization within the country. SWOT analysis is illustrated as a square part into four quadrants, each devoted to a component of SWOT. This visual overview provided a rapid indication of the leishmaniasis position. A complete list of issues was compiled to collect data related to each component of the SWOT. Several meetings were held to screen, categorize and weigh each component. The most related information and desired data about the disease management and control program were selected to analyze the internal and external factors presented in Supplementary Table 1. Hence, a scoring matrix of the internal and external factors directly and/or indirectly associated with the application of the control program was established, and subsequently, a final score of the internal and external factors was obtained (Figure 6). Based on the scoring matrix SWOT of Bryson (154), the overall position of the national leishmaniasis situation was calculated to be in the Strength/Opportunity (SO) position. This score indicates that the national leishmaniasis strengths and opportunities are already reasonably moderate. This situation allows us to firmly step towards its activities and targets in the future elimination program.

## 11. Discussion and conclusions

In the last decade, CL has considerably been expanded spatially, and numerous outbreaks and emerging epidemics of variable magnitudes occurred, attributed to the dynamic of the disease and provision of many confounding determinants such as environmental changes, climatic conditions, disasters (earthquakes and floods), migration, and population movement among many others. Despite a significant reduction in VL cases, the geographical range of CL has remarkably enlarged (12, 14, 55, 56, 86, 155, 156). The policy-makers have seriously overlooked the disease, and health authorities do not consider CL a severe threat and urgent public health concern in Iran and abroad. This is mainly due to the disease’s apparent non-serious and under-reporting nature, other confounding factors, and numerous chronic diseases. Iran is among the frontline-affected countries where many cases have officially been reported, although the actual number of new cases is assumed to be far beyond the reported statistics.

An important event in efforts against leishmaniasis was attained following the meeting held by WHO in 2007 (157); however, it underlined gains and challenges, eliciting further pledges from member states and private partners. Additional commitment is highly required to achieve the goals to control this neglected disease. Of the strategies recommended by the WHO, intensified CL management where effective treatment and confirmed vaccines are not yet available should be firmly targeted through better access to resources, organized cases detection, and decentralized clinical management to reduce morbidity and interrupt transmission (58).

Control of vector and reservoir hosts in an integrated manner combines multiple inter-sectoral interventions that enhance various measures, including efficacy, ecosystem approaches, and sustainability of disease control measures against sandflies and small gerbils. Lastly, improving socio-economic factors plays a critical role in achieving the goals. Documents compiled by the United Nations indicated that not only in Iran but also in the world as well, there are still 2.0 billion people who do not have basic sanitation facilities such as toilets or latrines (158). CL and all infectious diseases and several NTDs will not be eliminated and not eradicated until this situation improves. It is worth mentioning exploring and getting lessons from the VL regional leishmaniasis control programs in the Indian Subcontinent (159). During the past years, substantial efforts have been made, including capacity-building, strengthening improvement, accessibility to medicines and diagnostic tools, enhanced surveillance, monitoring, and evaluation.

In conclusion, analyses of the current epidemiological data indicate that Iran has made steady progress and remarkable advancement over the past decade, yet persistent challenges exist to reduce the CL burden in the country. Well-trained staff and experienced clinical practitioners must strengthen country-level capacity-building to sustain efficient control programs through the healthcare system (160). Cutaneous leishmaniasis is rooted in poverty; therefore, improving socioeconomic necessities toward basic living standards will ensure long-term control strategies and foster the elimination of the disease. A robust commitment to multidimensional approaches is critical to making advancements nationwide. This will require coordinated challenging activities through all governmental sections, including policy-makers, health professionals, clinical practitioners, senior researchers, private parties, and NGOs. Promotion of early case detection, prompt diagnosis, intensified effective treatment, increased access to available medicines, and capacity for environmental interventions on a long-term basis could hopefully facilitate the strategies and improve the prevention and control of not only CL but also neglected zoonotic diseases.

Furthermore, setting up early warning systems for CL can support forecast CL outbreaks. Hence, operational readiness and immediate response mechanisms should be in place in high-risk areas within endemic countries. However, the most decisive evidence gaps persist, and more innovative tools are critical before CL can eventually be controlled.

## Author contributions

IS and MG designed research and generated the study plan. FG, BA, MZ, SD, FS, AKh, MB, and AA performed the data collection in the field. IS, MG, AKh, MB, MN, OZ, and MS conducted the analysis and drafted the manuscript. IS, MG, MRF, AKa, ES, SA, and MB analyzed the data. IS, MG, OZ, AKh, MB, and MRS revised the manuscript. MG and IS had primary responsibility for final content. All authors contributed to the article and approved the submitted version.

## Funding

This work was supported by the Center for Diseases Control and Prevention, Communicable Diseases, Tehran, Iran (grant number: 202096276) and WHO Country Office.

## Conflict of interest

The authors declare that the research was conducted in the absence of any commercial or financial relationships that could be construed as a potential conflict of interest.

## Publisher’s note

All claims expressed in this article are solely those of the authors and do not necessarily represent those of their affiliated organizations, or those of the publisher, the editors and the reviewers. Any product that may be evaluated in this article, or claim that may be made by its manufacturer, is not guaranteed or endorsed by the publisher.
