# Supplementary figures and images for: Cutaneous leishmaniasis situation analysis in the Islamic Republic of Iran in preparation for an elimination plan
Source: Front Public Health. 2023 Apr 28;11:1091709. doi: 10.3389/fpubh.2023.1091709 (PMC10176454; doi:10.3389/fpubh.2023.1091709)

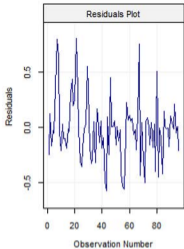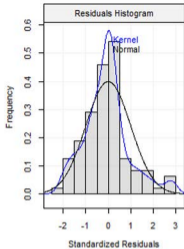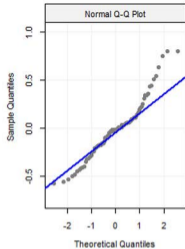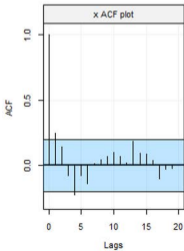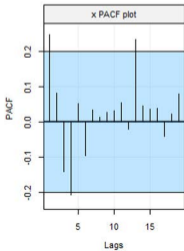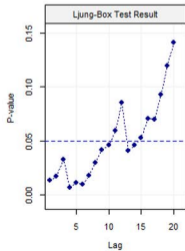

Supplement: Supplementary file 1 [file Data_Sheet_1.PDF]
