# Supplementary material for: Cutaneous leishmaniasis situation analysis in the Islamic Republic of Iran in preparation for an elimination plan
Source: Front Public Health. 2023 Apr 28;11:1091709. doi: 10.3389/fpubh.2023.1091709 (PMC10176454; doi:10.3389/fpubh.2023.1091709)
Supplement: Supplementary file 2 [file Data_Sheet_2.PDF]

TABLE S2 Analysis of internal and external factors

Strengths	Coefficient	Ranking	Score
1. Available treatment modalities in line with WHO strategy	6	3	18
2. Experienced health personnel and expert physicians at the district level in endemic areas	6	3	18
3. Expert staff and knowledgeable personnel	6	3	18
4. Registration of CL & VL at district hospitals	5	1	5
5. Wide use of screens and coolers in domestic houses	4	2	8
6. Integrated vector-control measures	3	2	6
7. Well-experienced staff to disseminate the research programs	2	2	4
8. Affiliated university and research center at district and/or provincial level	2	2	4
9. PHC network	5	2	10
10. The existence of a national and provincial Leishmaniasis committee	4	3	12
11. The existence of a defined monitoring system	3	3	9
12. Empowerment and valuable experiences in control programs, elimination or eradication of the infectious disease in Iran, including polio & malaria	6	3	18
**Weaknesses**	**Coefficient**	**Ranking**	**Score**

1. No routine follow-up of cases	6	2	12
2. Lack of GIS capacity linked to surveillance data	2	2	4
3. No formal and strategy for surveillance of vector and reservoirs (dogs and gerbils)	3	3	9
4. Lack of epidemiologists at the district level	3	2	6
5. Poor availability of diagnostic facilities	3	2	6
6. No early detection of cases	3	2	6
7. Lack of drug of choice	3	4	12
8. Poor drug adherence (due to various constraints)	3	3	9
9. No responsible dermatologist at the district level	3	3	9
10. No routine follow-up assessment of cases	3	3	9
11. Lack of knowledge of diagnostic and therapeutic updates	3	3	9
12. Lack of a web-based information-sharing system to disseminate technical information and exchange	4	4	16
13. Weakness in teamwork	3	3	9
14. Lack of financial resources	3	3	9
15. Lack of active participation of private sector doctors in the disease reporting system	3	3	9
**Total**	100		264

Scoring matrix of the external factors			
**Opportunities**	**Coefficient**	**Ranking**	**Score**
1. Potential of GIS application at the district level	4	3	12
2. Provision of more effective drugs	3	3	9
3. Inter-sectoral collaborations	5	4	20
4. Availability of qualified human resources	5	4	20
5. NIMAD supports operational research projects at the national level	3	3	9
6. Development in high-risk areas	5	3	15
7. Collaboration with non-governmental organizations (NGOs)	3	3	9
8. Coverage and proper IT development	5	4	20
**Threats**	**Coefficient**	**Ranking**	**Score**
1. Patients referred to private healthcare facilities (missing records)	4	4	16
2. Fund limitation	5	4	20
3. New emerging foci of CL	5	4	20
4. Lack of control approaches against gerbils and dogs	6	4	24
5. Keeping dogs and carnivores at home	5	4	20
6. Poor stray dogs management	4	4	16
7. Resistance to prevalent insecticides	3	3	9

8. Retirement of experienced health personnel	3	3	9
9. Research projects are primarily focused on only knowledge rather than on practical aspects (interventional research)	2	2	4
10. Unauthorized border movement (migration)	4	4	16
11. Economic sanctions of the country	5	4	20
12. The existence of vulnerable strata and marginalization	7	4	28
13. Unpredictable disasters	2	2	4
14. Populations of nomads	5	4	20
15. Poor health culture in the marginalized area	7	4	28
**Total**	100		368
